# Virtual screening, molecular docking studies and DFT calculations of FDA approved compounds similar to the non-nucleoside reverse transcriptase inhibitor (NNRTI) efavirenz

**DOI:** 10.1016/j.heliyon.2020.e04642

**Published:** 2020-08-11

**Authors:** Maryam A. Jordaan, Oluwakemi Ebenezer, Nkululeko Damoyi, Michael Shapi

**Affiliations:** Faculty of Natural Science, Department of Chemistry, Mangosuthu University of Technology, 511 Mangosuthu Highway, Durban, 4000, South Africa

**Keywords:** Pharmaceutical chemistry, Theoretical chemistry, COVID-19, NNRTI, Virtual screening, Efavirenz, HIV

## Abstract

Severe acute respiratory syndrome coronavirus 2 (SARS-CoV-2) was confirmed as the causative virus of COVID-19 disease, which is currently a worldwide pandemic. Efavirenz, a non-nucleoside reverse transcriptase inhibitor (NNRTI), is one of the most potent chemical compounds proposed to treat COVID-19 infection. We, therefore, performed virtual screening on FDA approved drugs that are similar to the efavirenz moiety. Subsequently, the compounds were subjected to screening by analyzing their drug-likeness, such as Lipinski's rule of five and ADMET properties. Molecular docking study revealed that Met165, His41, His163, and Phe140 were important interacting residues for COVID-19 main protease receptor-ligand interaction. Five top-ranked compounds, podophyllotoxin, oxacillin, lovastatin, simvastatin, and gefitinib, were selected by virtual screening and docking studies. The highest occupied molecular (HOMO) orbital, lowest unoccupied molecular orbital (LUMO) and energy gap values was calculated using density functional theory (DFT). The results of the study showed that lovastatin and simvastatin might be considered as lead compounds for further development for COVID-19 main protease inhibitors.

## 1 Introduction

**Figure 1:**
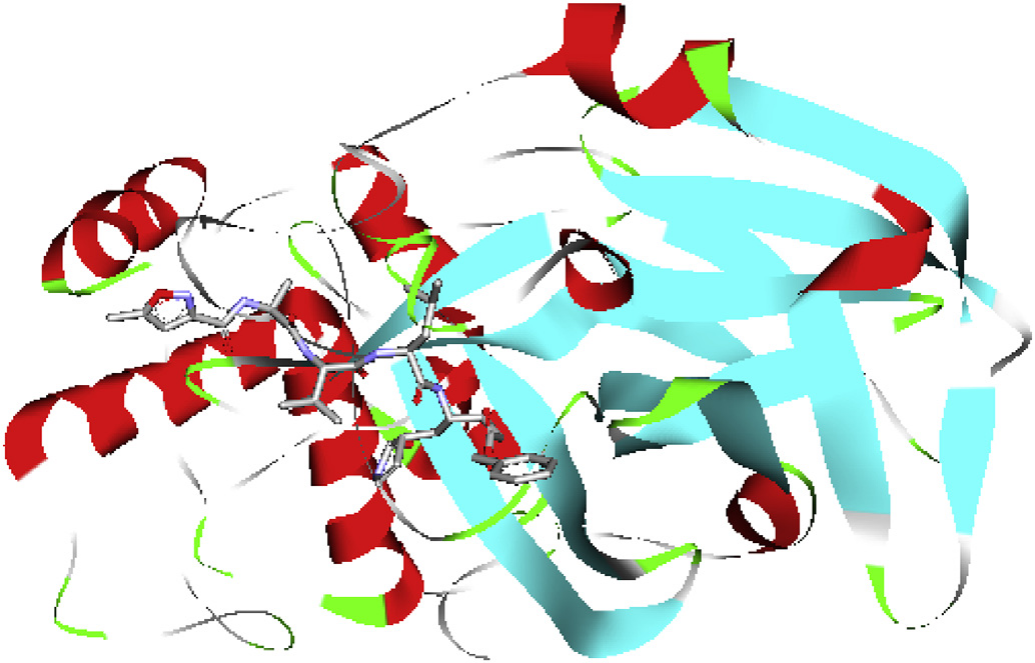
The model structure of the COVID-19 main protease in complex with a peptidomimetic inhibitor (PDB; 6LU7).

The novel coronavirus (COVID-19)was identified in the Hubei Province of China in 2019, and, today, over half a million people are currently infected globally with more than 20 834 deaths [1, 2, 3]. Coronaviruses are positively stranded RNA viruses that cause respiratory, enteric, and central nervous system diseases. The recent novel COVID-19 virus is considered a betacoronavirus alongside severe acute respiratory syndrome coronavirus (SARS-CoV) and the Middle East respiratory syndrome (MERS-CoV). More also, additional sequence alignment revealed a 96.1% comparison to the sequence of the main protease between COVID-19 and SARS-CoV [4, 5]. The crystal structure of the COVID-19 main protease in complex with a peptidomimetic inhibitor (PDB code 6LU7) is presented in (Figure 1). Viruses like the COVID-19 mutate rapidly rendering it difficult to design an appropriate treatment. Previous studies demonstrated that the main protease of SARS-CoV is essential for the life cycle of the virus, and considered to be an attractive target for drug development [4, 6]. Drugs that target conservative protease are usually capable of preventing the replication and proliferation of the virus while reducing the risk of mutation mediated drug-resistance [2, 4].

Recent positive data highlights the application of a cocktail of antivirals, including two antiretroviral components lopinavir and ritonavir, by doctors in Thailand who were able to cure a COVID-19 patient using this treatment. These two antiretroviral components are protease inhibitors designed to block HIV viral replication and holds that these drugs could do the same for COVID-19 [7, 8].

**Figure 2:**
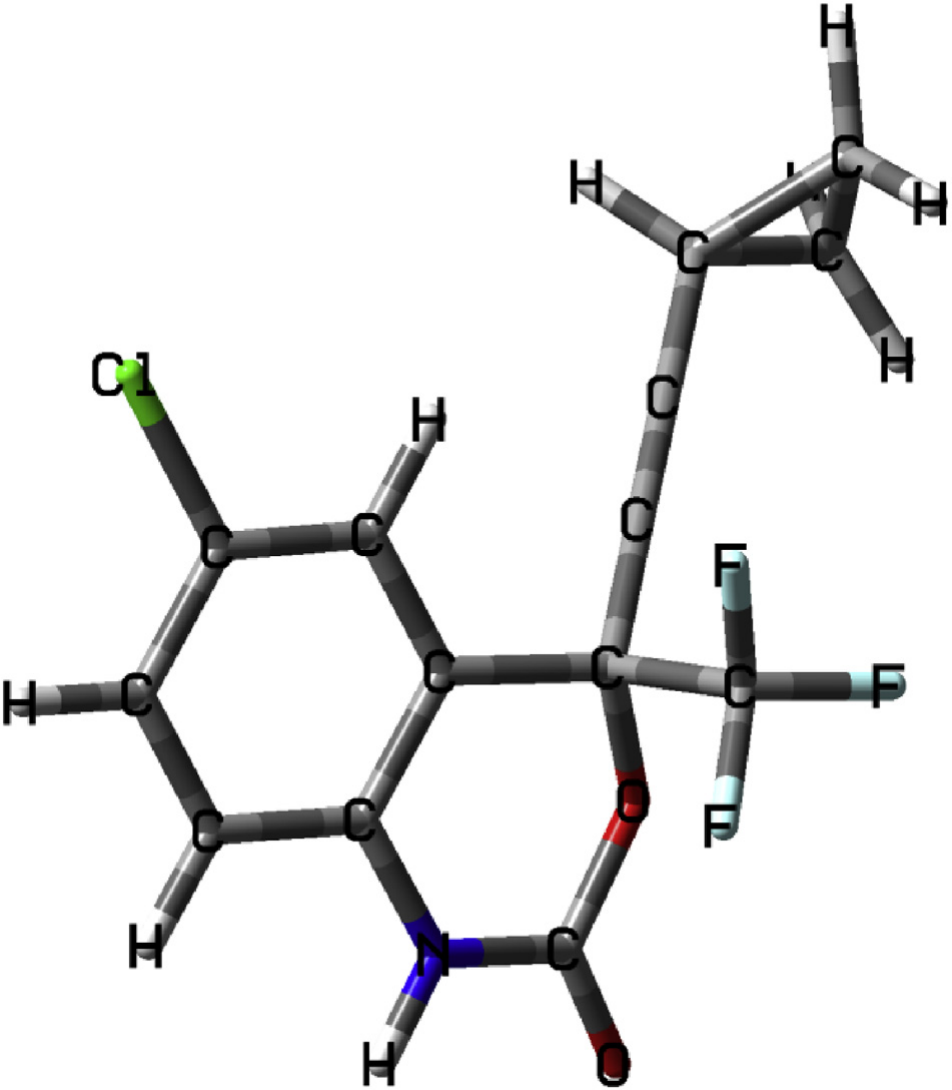
Optimized structure of efavirenz.

Furthermore, a similar study conducted by Beck and co-workers (2019) highlighted the application of a Molecule Transformer-Drug Target Interaction (MT-DTI) model to identify possible existing compounds to treat COVID-19. They predicted antiretroviral medication for HIV as the best chemical compounds to treat COVID-19, i.e.: atazanavir, efavirenz, ritonavir, and dolutegraviran [9]. Shaha and coworkers also identified a range of ARVs, including efavirenz see Figure 2 as a potential treatment for COVID-19 [10]. Confirming ARVs as a potential therapy, Yan Li and his team at the Sichuan University and Army Medical University in China (2020) identified, *via* a large chemical screening, four molecular drugs with high-affinity to a coronavirus protein. Of these, two were ARVs, i.e. bictegravir and nelfinavir. The other two were Prulifloxacin, a chemotherapeutic antibiotic, and Tegobuvi, an antiviral drug used in the treatment of Hepatitis C infection [4]. Contini (2020) also used virtual screening and identified four ARVs to treat COVID-19, i.e. indinavir, lopinavir, and atazanavir and cobicistat [2, 11, 12, 13, 14, 15, 16]. Furthermore, three types of *in silico* DTI prediction methods are used, i.e. molecular docking, similarity-based, and deep learning-based [11, 17]. The selection of the efavirenz scaffold was based on the following factors: firstly, ARVs have been utilized as an antiviral regime for patients infected with the coronavirus and secondly, *in silico* DTI predictions identified antiretroviral (ARV), efavirenz, a non-nucleoside reverse transcriptase inhibitor (NNRTI) as one of the most potent chemical compounds with the inhibitory potency with *Kd* of 199.17 nM against the COVID-19 main protease [9].

In this study, virtual screening, also called *in silico* screening, was chosen to provide a rapid and inexpensive method for the discovery of FDA approved active compounds exhibiting a scaffold similar to efavirenz, which binds to the active pocket of COVID-19 main protease. Virtual screening and molecular docking results revealed promising potential hit compounds for COVID-19 main protease inhibition. Density functional theory (DFT) was used further to calculate the orbital energy value and the energy gap.

## 2 Materials and methods

### 2.1 Ligand preparation

The active compounds similar to efavirenz were retrieved from the zinc database [18]. Protocol constraints such as biogenic data, FDA approval, anodyne, and sell data were set to filter out the compounds. A total of 232 molecules were retrieved and prepared for docking by subjecting to energy minimization using the Open Babel module in PyRx program.

### 2.2 Receptor preparation

The crystal structure of COVID-19 main protease (PDB: 6lu7) and the native ligand was downloaded from RCSB with a resolution of 2.16 Å [19]. Discovery Studio Visualizer software was used to prepare the receptor for docking. The native ligand and water molecules were deleted from the crystal structure of COVID-19 main protease. Molecular docking was performed with AutoDock 4.2 module implemented in PyRx 0.8 using the empirical free energy force field and Lamarckian genetic algorithm conformational search with the default parameters [20]. The grid on the ligand-binding site of the protein was centered at the binding site of X = -20, Y = 13, Z = 47, and the grid dimensions were 40 × 30 × 62 Å^3^. For further analysis, 43 compounds with a lowest binding affinity (<-7.0 kcal/mol) were selected. Protein-ligands interaction was analyzed using Discovery Studio Visualizer software.

### 2.3 Physicochemical and ADME biochemical prediction

Selected compounds from the molecular docking analysis were evaluated for their drug-like behavior through analysis of pharmacokinetic parameters required for absorption, distribution, metabolism, and excretion (ADME). QikProp module and Lipinski's rule integrated into virtual screening workflow (filtering option) of Schrödinger software was employed for calculations. We discarded 20 compounds, which were predicted to violate Lipinski's rule of five and, also fall out of optimum range for partition coefficient (QPlogPo/w), critical for estimation of absorption within the body; cell permeability (QPPCaco), a key factor governing drug metabolism and its access to biological membranes; QPPMDCK and percentage human oral absorption. Hence, 27 compounds were observed to have physicochemical and pharmacokinetic parameters within the acceptable range.

### 2.4 Density functional theory

Density functional theory (DFT) is a computational quantum mechanical modeling method used to examine the electronic structure and also to investigate the interactions involved between the receptors and the ligands. The electronic and structural properties of the five best hit compounds were calculated using the Becke3-Lee-Yang-Parr (B3LYP) method with the 6–31G(d,p) basis set aided by Gaussian 09. The calculated parameters used in this study include the highest occupied molecular orbital (HOMO) and the lowest unoccupied molecular orbital (LUMO) energies, electron affinity, and electrophilicity index. The molecular electrostatic potential surfaces (MEPs) were obtained from the population analysis calculations and visualized using Gauss View. These parameters play an influential role in explaining the magnitude of ligands interaction in the binding pocket of COVID-19 main protease.

### 2.5 Molecular dynamics

To validate the stability of the hit compound, we have performed molecular dynamics simulation using NAMD full setup through the MDWeb interface. The simulation process includes cleaning of the protein structure; fixing of side chains; addition of hydrogen atoms; neutralization, the addition of a solvent box and heating solvent to 300 K; reducing the restraints to just the protein backbone and minimization and equilibration of the system to finally achieve the structure prepared by simulation. To achieve a dry trajectory for the simulated protein, water molecules and ions were removed from the system.

## 3 Results and discussion

To validate our docking protocol, we redocked native ligand and the selected 27 compounds, into the binding pocket of COVID-19 main protease using AutoDock 4.2 module in PyRx tool with default parameters. Moreover, the binding affinity of the native ligand was selected as a benchmark. Five of these compounds exhibited superior binding affinity as well as good interaction compared to efavirenz and the native ligand (-6.5 and -7.4 kcal/mol respectively), thus selected for visual analysis. Refer to Table 1 for full data.

**Table 1:** Physicochemical and ADMET descriptors of efavirenz and the 27 dock compounds calculated from QikProp.

Molecule	Binding Affinity	MW	HBD	HBA	QPpolrz	QPlogPC16	QPlogPoct	QPlogPw	QPlogPo/w
Picato	-7.0	430.54	3	8.15	43.941	12.43	23.41	13.578	3.235
Gefitinib	-7.7	446.908	1	7.7	44.759	13.302	20.35	10.804	4.355
Eht0201	-5.6	278.31	0	7	29.044	8.317	13.427	8.177	1.176
Dicloxacillin	-7.2	470.326	1.25	7.75	40.522	12.4	20.394	14.035	2.52
Oxacillin	-7.8	401.436	1.25	7.75	39.907	12.236	20.481	15.401	2.364
Simvastatin	-7.8	418.572	1	6.7	45.909	12.849	20.603	9.457	4.67
Lovastatin	-7.6	404.545	1	6.7	43.257	12.147	19.716	9.281	4.252
Hyoscine	-7.2	303.357	0	6.7	30.356	9.009	13.285	8.03	1.247
(*S*)-Colchicine	-7.3	399.443	1	7.5	39.735	10.7	18.511	11.646	2.586
(*R*)-Colchicine	-7.3	399.443	1	7.5	39.735	10.7	18.511	11.646	2.586
Podophyllotoxin	-7.9	414.411	1	8.45	37.819	10.369	18.665	11.276	2.463
Pentamidine	-6.5	340.424	6	4.5	35.113	14.334	23.426	15.09	2.564
Altol	-6.1	266.339	4	6.45	28.471	10.176	18.409	16.261	0.172
Epoprostenol	-6.7	352.47	3	6.15	37.05	12.449	19.825	10.511	3.645
Pgx	-6.6	352.47	3	6.15	36.611	12.353	19.839	10.517	3.528
Urso	-7.2	392.578	3	5.4	40.054	11.825	20.873	10.957	3.785
Chenodal	-7.2	392.578	3	5.4	39.956	11.776	21.404	10.922	3.8
Degalol	-7.1	392.578	3	5.4	40.005	11.788	20.906	10.933	3.802
Cholic acid	-6.9	408.577	4	7.1	39.864	12.316	23.381	14.035	2.897
Enalaprilat	-7.2	348.398	3	8.5	36.266	12.203	22.246	15.652	-0.722
Piceid	-7.2	390.389	6	10.75	35.388	14.14	26.981	21.618	0.178
Pfizerpen	-7.0	334.389	1.25	6.25	34.46	10.833	16.853	15.782	1.83
Sufentanil	-6.6	386.551	0	6.7	43.447	12.783	17.658	8.379	4.055
Cephalexin	-6.7	347.388	3.25	7.25	34.128	11.614	20.635	16.746	-1.256
Ampicilin	-7.3	349.404	3.25	7.25	34.803	11.777	21.321	19.022	-1.946
Omeprazole	-6.6	345.415	1	8	35.293	10.448	17.167	13.818	2.232
Prazosin	-7.3	383.406	2	8	40.055	11.866	21.002	13.528	2.659
Efavirenz	-6.5	315.679	1	3.5	28.899	7.837	13.636	6.799	3.516

Molecule	QPlogHERG	QPPCaco	QPlogBB	QPPMDCK	QPlogKp	QPlogKhsa	HumanOralAbsorption
Picato	-4.186	628.425	-0.975	299.423	-3.152	0.4	3
Gefitinib	-7.165	1049.826	0.307	2306.299	-2.681	0.367	3
Eht0201	-4.416	495.631	-1.037	231.661	-3.362	-0.721	3
Dicloxacillin	-0.661	22.25	-1.002	68.544	-4.468	-0.223	2
Oxacillin	-1.777	15.896	-1.43	19.858	-4.245	-0.355	2
Simvastatin	-4.736	652.454	-1.067	311.817	-2.822	0.783	1
Lovastatin	-4.495	869.722	-0.902	425.43	-2.58	0.611	3
Hyoscine	-4.396	440.051	0.029	225.371	-4.02	-0.611	3
(*S*)-Colchicine	-3.08	927.454	-0.599	710.539	-2.527	-0.086	3
(*R*)-Colchicine	-3.08	927.448	-0.599	710.537	-2.527	-0.086	3
Podophyllotoxin	-3.94	1528.112	-0.418	782.348	-2.391	-0.075	3
Pentamidine	-6.671	83.493	-2.728	33.789	-7.263	-0.172	2
Altol	-4.478	37.094	-1.169	33.983	-5.18	-0.752	2
Epoprostenol	-3.586	41.239	-2.35	20.05	-3.668	0.052	2
Pgx	-3.461	34.615	-2.403	16.592	-3.828	0.031	2
Urso	-2.092	43.15	-1.5	21.056	-4.373	0.451	3
Chenodal	-2.052	47.21	-1.453	23.205	-4.297	0.447	3
Degalol	-2.114	46.796	-1.472	22.985	-4.304	0.447	3
Cholic acid	-2.046	26.197	-1.757	12.278	-4.698	0.119	2
Enalaprilat	-0.948	2.211	-1.324	1.475	-5.56	-0.876	1
Piceid	-5.723	34.353	-2.729	12.939	-4.199	-0.742	2
Pfizerpen	-0.36	17.716	-1.189	41.948	-3.568	-0.742	2
Sufentanil	-6.823	1666.192	0.398	1627.984	-2.13	0.257	3
Cephalexin	-2.545	3.129	-1.359	3.188	-6.505	-0.631	2
Ampicilin	-1.101	2.153	-1.227	4.537	-6.339	-0.909	1
Omeprazole	-5.174	59.206	-0.532	951.724	-1.969	-0.242	2
Prazosin	-5.709	891.968	-0.802	437.204	-2.371	0.061	3
Efavirenz	-4.343	1524.372	0.075	6408.891	-2.535	0.267	3

M.W – molecular weight; log S_*wat*_ - aqueous solubility (-6.5–0.5); log *K*_*HSA*_ - logarithm of predicted binding constant to human serum albumin (-1.5–1.5); QPlogPw – water/gas partition (4.0–45.0); QPlogPC16 –hexadecane/gas partition (4.0–18); log BB - logarithm of predicted blood/brain barrier partition coefficient (-3.0-1.2); Caco-2 - cell membrane permeability (<25 poor >500 good); HBA - number of hydrogen bond acceptors (2–20); HBD number of hydrogen bond donors (0–6); QP_*polrz*_ - predicted polarizability (13–70); log *HERG* the predicted IC_50_ value for the blockage of HERG K^+^ channels (concern below -5); QPPMDCK – predicted MDCK cell permeability in nm/sec (<25 poor >500 great); log *K*_p_ - predicted skin permeability and 95% of drugs: (-8 to -1);, log *K*_*HSA*_ - logarithm of predicted binding constant to human serum albumin (-1.5–1.5), Human Oral Absorption – 1-low, 2-medium, 3- high.

The five selected compounds include simvastatin and lovastatin, HMG-CoA reductase inhibitor; oxacillin, a penicillinase-resistant β-lactam; podophyllotoxin, which is an antimitotic; and Gefitinib an epidermal growth factor receptor (EGFR) inhibitor [21, 22, 23, 24, 25, 26]. The identification of the key contributing residues in the binding pocket of the C19MP was performed using the Discovery Studio Visualizer.

**Figure 3:**
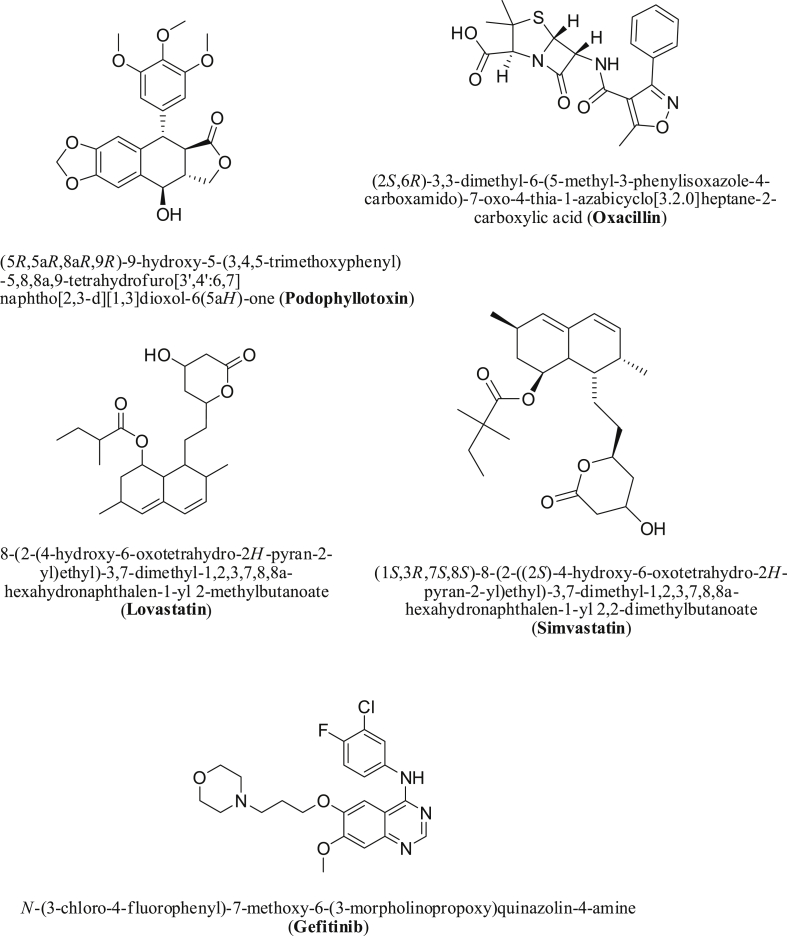
Structures of the FDA approved active compounds exhibiting a scaffold similar to efavirenz.

**Figure 4:**
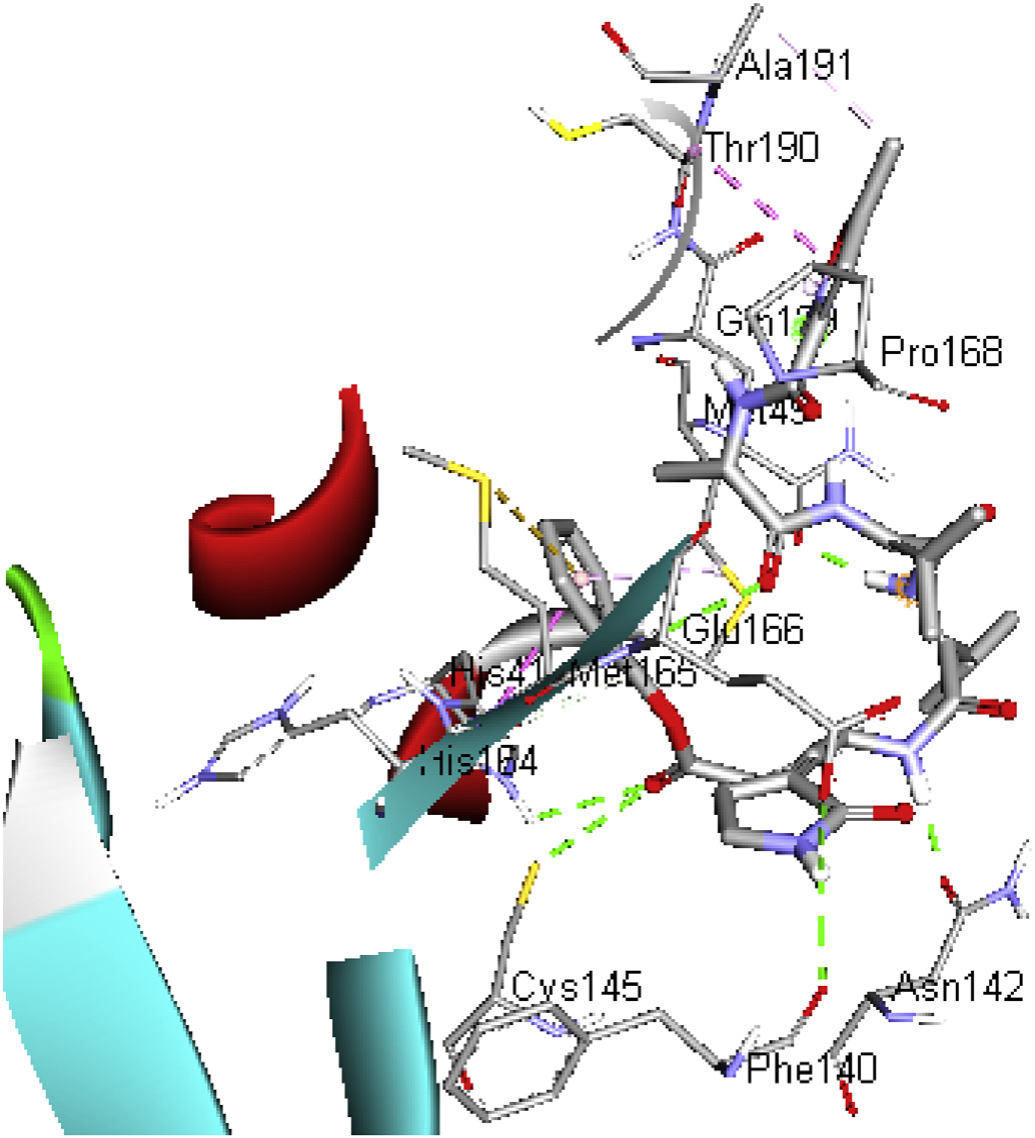
Schematic representation of C19MP (PDB: 6LU7) interactions with the native ligand  protein  ligand  hydrophobic interaction  carbon-hydrogen bond.

The docked results showed that inhibitors swing between the space of hydrophobic residues; Met165, His41, Met49, Cys145, His165 and Leu27 which gave more conformational freedom, on the one hand; residues Glu166, His163, Thr26, Phe140, Thr190, His164 and Gln189, Gln143 showed hydrogen bond interaction with the ligands. Also, protein-ligands interaction showed that the presence of naphthalene, quinazoline, isoxazole, benzyl, and tetrahydropyran rings played an important role. The details of the structures with the best docking scores are provided in Figures 3 and 4.

**Figure 5:**
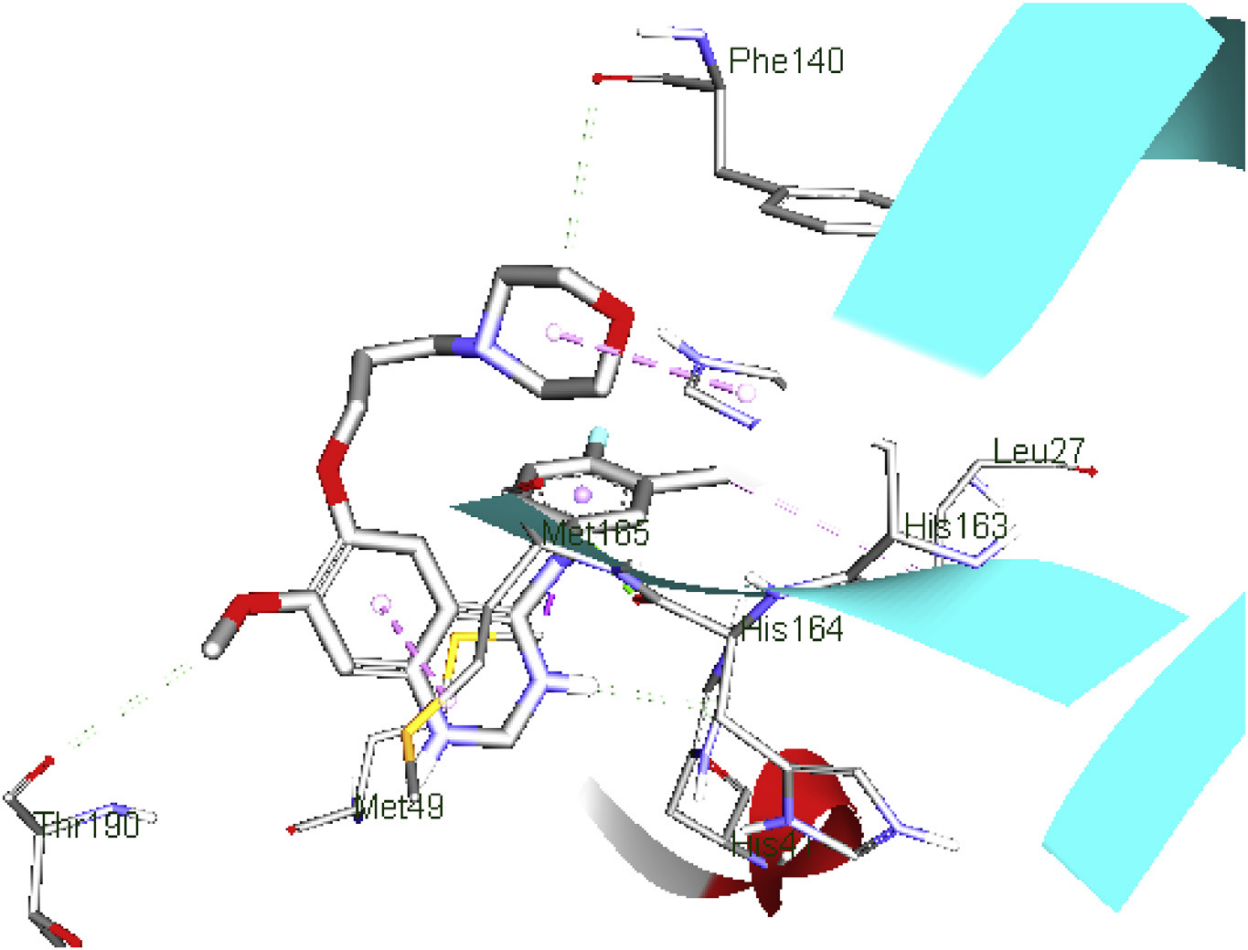
Schematic representation of C19MP (PDB: 6LU7) interactions with gefitinib. protein  ligand  hydrophobic interaction  carbon-hydrogen bond.

The quinazoline ring of gefitinib (b.e = 7.7 kcal/mol) formed π-alkyl interaction with Met165; meanwhile, one of the *N* atom donors in the ring formed a conventional hydrogen bond with the *O* atom acceptor of conserved residue His41, with bond distance (b.d) of 2.50 Å. Besides, the amine group formed a conventional hydrogen bond with His164 (b.d = 2.66 Å and bond angle, (b.a) = 131.02^o^ and 153.7^o^ respectively). Carbon hydrogen bonds are formed between the morphine ring, methyl group and, residue Phe140 and Thr190 (b.d = 3.69, and 3.57 Å; b.a = 103.12 and 97.39^o^ respectively). Apart from H-bonding, the *Cl* atom at the R_5_ position of the benzyl ring formed hydrophobic interaction with Leu27. These hydrophobic and H-bonding interactions of C19MP with the ligand may account for the good binding affinity (Figure 5).

**Figure 6:**
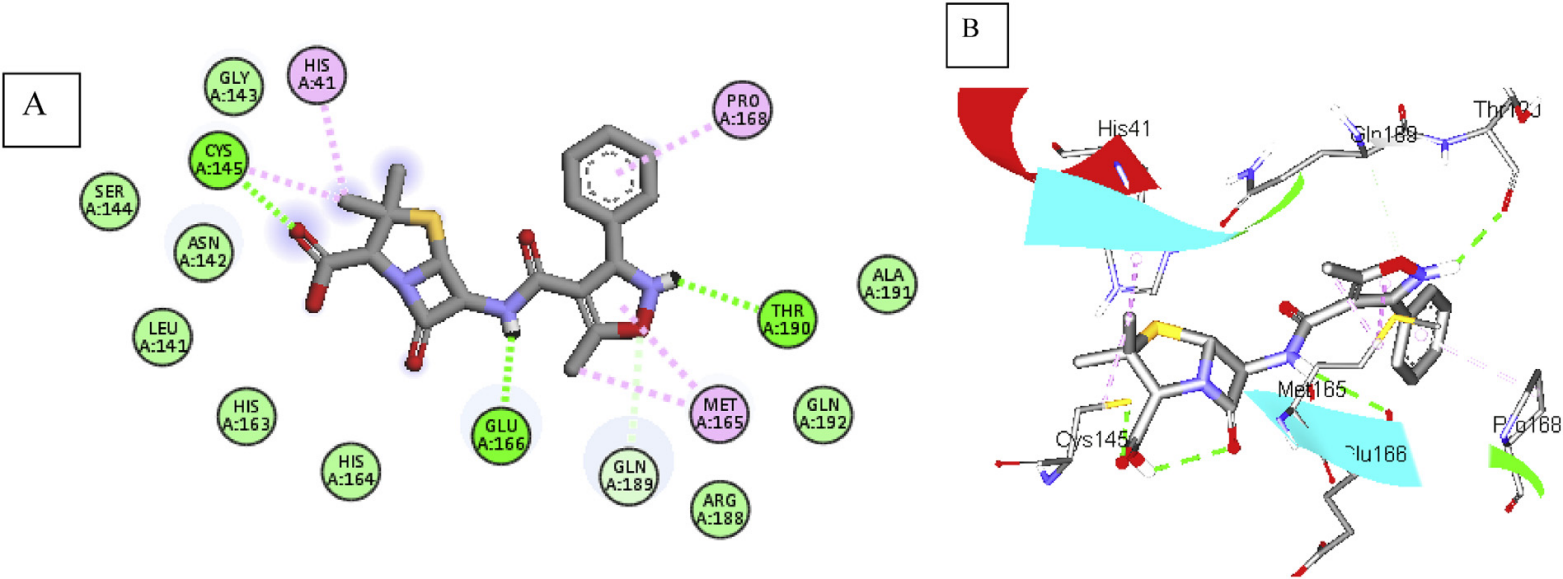
2D representation (**A**) and 3D representation (**B**) of oxacillin in the binding pocket of C19MP (PDB: 6LU7)  protein  ligand  hydrophobic interaction  carbon-hydrogen bond.

The amine group and the *O* atom of the isoxazole ring (oxacillin, b.e = 7.8 kcal/mol) exhibit H-bond with Thr190 and Gln189 (b.d = 2.32 and 3.40 Å; b.a = 137.72, 144.5 and 91.7^o^ respectively). Meanwhile, Met165 was sandwiched between the isoxazole ring and the methyl substituent (Figure 6B). The phenyl group attached to the isoxazole ring interacts hydrophobically with Pro168 also contributes to the stabilization of the complex. Other residues like Ala19, Gln192, Agr188, Gln189, His164, 163, Gly143, Ser144, and Leu141 make close contact with the ligand without interactions (Figure 6A).

Moreover, His41 and Cys145 interacted with methyl group substituent, which was accompanied by one conventional H-bonding between the carboxyl oxygen atom and Cys145 (b.d = 3.55 Å; b.a = 107.66 and 108.0^o^ respectively), whereas the amine linker horizontally formed H-bond with Glu166 (b.d = 2.70 Å, b.a = 128.93, 117.4^o^).

In the docked complex of lovastatin (b.e = 7.6 kcal/mol), the *O* atom of tetrahydropyran moiety formed a conventional H- bond with *N* atom of Glu166 (b.d = 2.64 Å, b.a = 111.03 and 104.82^o^ respectively), the carbonyl oxygen atom interact with the *N* atom of His163 *via* H-bonding (b.d = 2.13 Å and b.a = 115.19 and 142.06^o^ respectively), meanwhile the secondary hydroxyl group exhibited an H-bond network with Phe140 (b.a = 2.82 Å, b.a = 135.37 and 120.80^o^, respectively).

**Figure 7:**
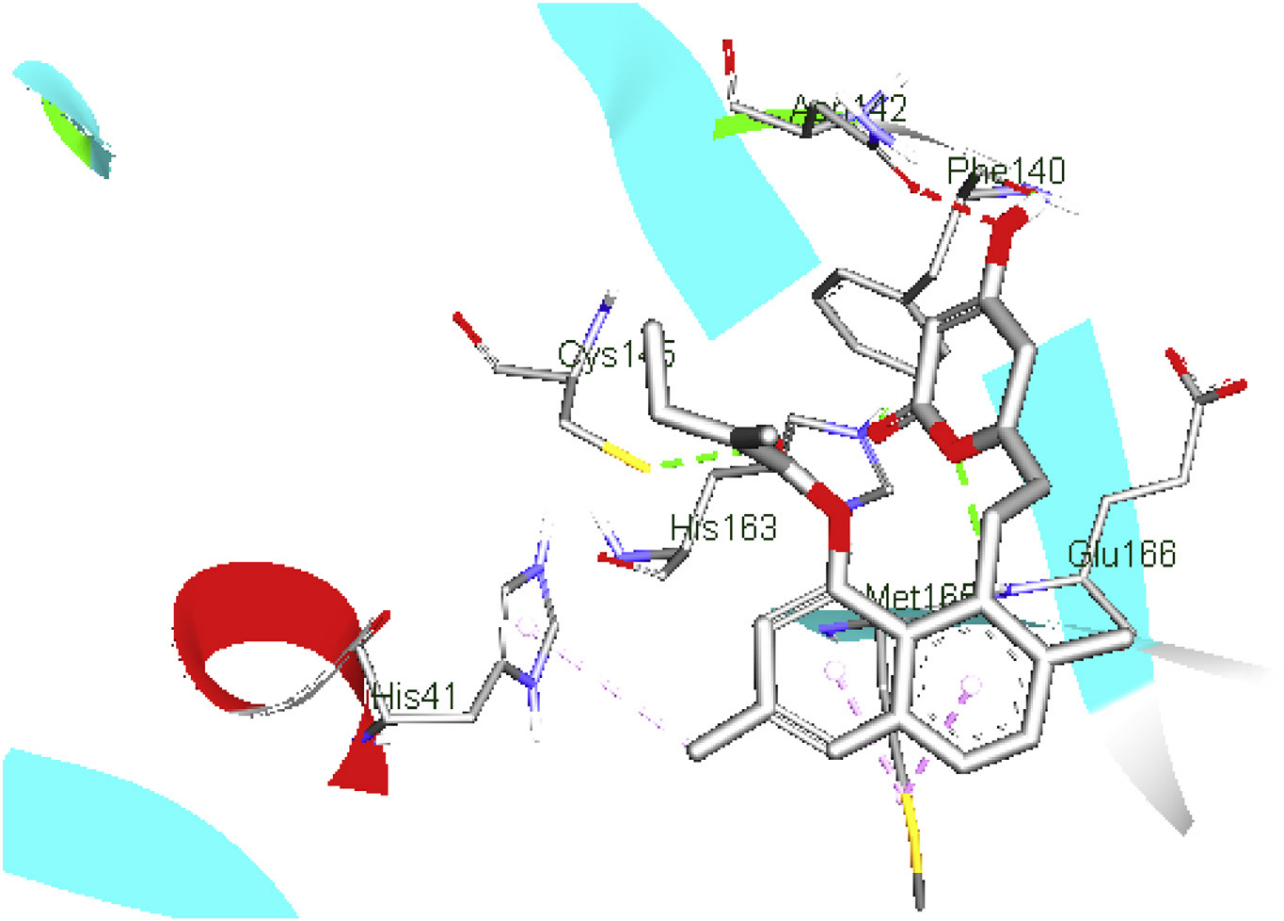
Schematic representation of COVID-19 main protease (PDB: 6LU7) interactions with lovastatin protein  ligand  hydrophobic interaction  hydrogen bond.

The naphthalene ring and the methyl group, which is adjacent to methyl butanoate, formed π-alkyl interaction with the side chain of Met165 and His41; the carbonyl oxygen group of methyl butanoate formed H-bonding interaction with the side chain of Cys145. From the above results, it can be pointed out, that H-bond interactions with the key binding residues Gln166, His163, Phe140, and Cys145 are major motivators for the stabilization of the inhibitor within the catalytic pocket. In contrast, hydrophobic interactions played a minor role (Figure 7).

**Figure 8:**
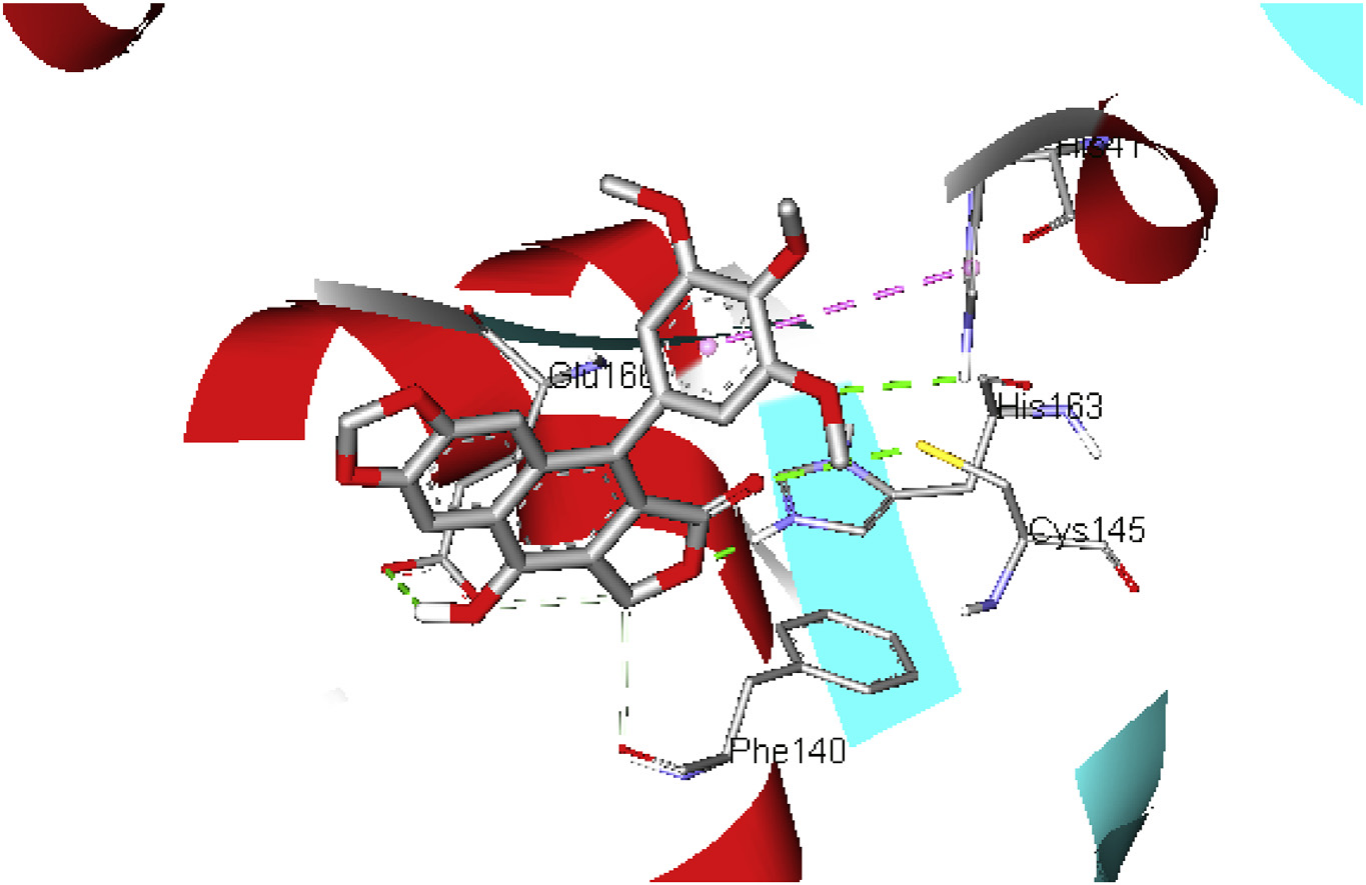
Schematic representation of COVID-19 main protease (PDB: 6LU7) interactions with podophyllotoxin. protein  ligand  hydrophobic  interaction hydrogen bond.

The *O* atom, carbonyl oxygen group, as well as Sp^3^ hybridized carbon in the furanone ring (podophyllotoxin, b.e = 7.7 kcal/mol)**)**, interact with residue His163, Cys145, and Phe140 via H-bond (b.d = 1.91, 3.45, and 3.20 Å; b.a = 99.1, 157.3 and 108.0^o^, respectively. The imidazole ring of conserved residue His41 was sandwiched between the phenyl ring and the *meta*-OCH_3_ substituent on the phenyl ring; this was accompanied by one hydrophobic interaction. These interesting interactions potentially deepen the binding strength of podophyllotoxin to the C19MP receptor and seem to be critical (Figure 8).

**Figure 9:**
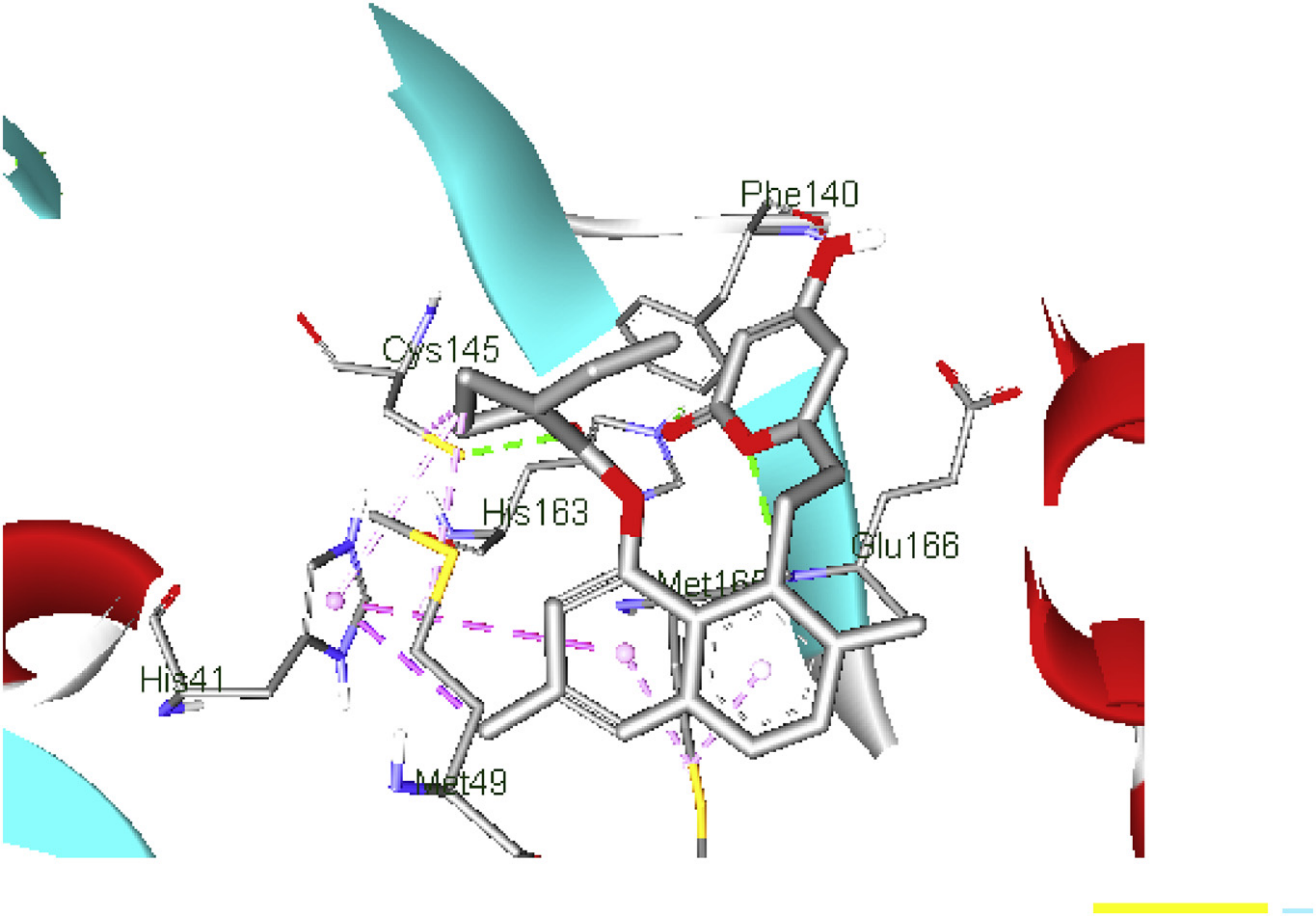
Schematic representation of C19MP (PDB: 6LU7) interactions with simvastatin.  protein  ligand  hydrophobic interaction  hydrogen bond.

A binding affinity score of −7.8 kcal/mol was obtained for the docking of simvastatin in the binding site of C19MP (Figure 9) refer to supporting information, which also indicates strong interactions between the ligand and the receptor. The carbonyl oxygen group and the *O* atom in the tetrahydropyran ring interact with His163 and Glu166 via H-bond (b.d = 2.21, 2.67 Å; b.a = 115.6, 125.1^o^, respectively). The naphthalene group extends into the hydrophobic pocket potentiating the ligand-receptor interaction (His 41 and Met165); the ring stacks vertically with the imidazole ring of His41 forming a π-π T-shaped interaction. Additional stability to this complex is afforded by the formation of a hydrogen bond interaction between the carbonyl oxygen atom of dimethylbutanoate and residue Cys145.

The presence of a dimethyl substituent also assists with strong binding inside the active site of C19MP by forming hydrophobic interactions with residues His41, Cys145, and Met49, respectively. Physicochemical and ADMET descriptors of the five best dock compounds are shown in Table 2.

**Table 2:** Physicochemical and ADMET descriptors of the six best dock compounds from QikProp.

Zinc Id	mol_MW	HBD	HBA	QPpolrz	QPlogPC16	QPlogPoct	QPlogPw	QPlogPo/w	QPlogS
Gefitinib	446.908	1	7.7	44.759	13.302	20.35	10.804	4.355	-5.261
Oxacillin	401.436	1.25	7.75	39.907	12.236	20.481	15.401	2.364	-3.365
Lovastatin	404.545	1	6.7	43.257	12.147	19.716	9.281	4.252	-5.796
Podophyllotoxin	414.411	1	8.45	37.819	10.369	18.665	11.276	2.463	-3.541
Simvastatin	418.572	1	6	45.909	12.849	20.603	9.457	4.67	-7.058

Zinc Id	QPlogHERG	QPPCaco	QPlogBB	QPPMDCK	QPlogKp	QPlogKhsa	Human Oral Absorption
Gefitinib	-7.165	1049.83	0.307	2306.299	-2.681	0.367	3
Oxacillin	-1.777	15.896	-1.43	19.858	-4.245	-0.355	2
Lovastatin	-4.495	869.722	-0.902	425.43	-2.58	0.611	3
Podophyllotoxin	-3.94	1528.11	-0.418	782.348	-2.391	-0.075	3
Simvastatin	-4.736	652.454	-1.067	311.817	-2.822	0.783	1

M.W – molecular weight; log S_*wat*_ - aqueous solubility (-6.5–0.5); log *K*_*HSA*_ - logarithm of predicted binding constant to human serum albumin (-1.5–1.5); QPlogPw – water/gas partition (4.0–45.0); QPlogPC16 –hexadecane/gas partition (4.0–18); log BB - logarithm of predicted blood/brain barrier partition coefficient (-3.0-1.2); Caco-2 - cell membrane permeability (<25 poor >500 good); HBA - number of hydrogen bond acceptors (2–20); HBD number of hydrogen bond donors (0–6); QP_*polrz*_ - predicted polarizability (13–70); log *HERG* the predicted IC_50_ value for the blockage of HERG K^+^ channels (concern below -5); QPPMDCK – predicted MDCK cell permeability in nm/sec (<25 poor >500 great); log *K*_p_- predicted skin permeability and 95% of drugs: (-8—1); log *K*_*HSA*_ - logarithm of predicted binding constant to human serum albumin (-1.5–1.5), Human Oral Absorption – 1-low, 2-medium, 3- high.

**Figure 10:**
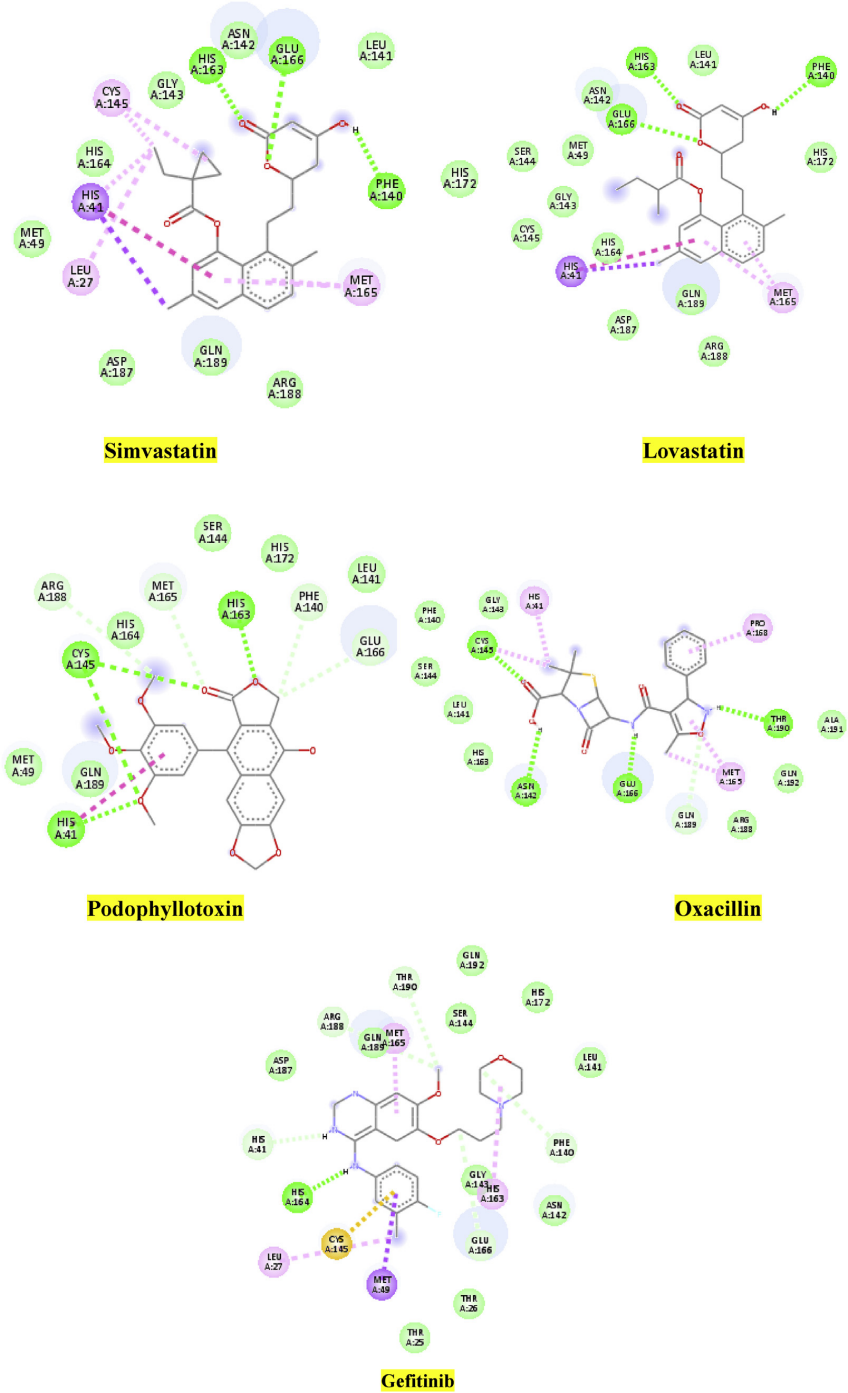
2D representation of the hit compounds in the binding pocket of C19MP after molecular dynamics (PDB: 6LU7)  protein  ligand  hydrophobic interaction  carbon-hydrogen bond.

Furthermore, to validate the docked results of the hit compounds, we performed MD simulations on the COVID-19 main protease and re-docked all the five compounds. The re-docked results revealed an increase in the binding affinity of simvastatin and lovastatin (8.2 and 7.9 kcal/mol), respectively. Surprisingly, there was a decrease in the binding affinity of podophyllotoxin (7.7 kcal/mol), and the binding affinity of oxacillin and gefitinib (7.7 kcal/mol) remained constant. Interestingly**,** the ligands were bound inside the active site of a similar pose and also formed interaction with more residues, mainly through H-bonding Figure 10.

### 3.1 Density functional theory

Frontier molecular orbitals of five-hit compounds specify a crucial role of charge-transfer interactions with the binding site of COVID-19 main protease. The higher HOMO value denotes a molecule with a good electron donor, whereas a lower value implies a weak electron acceptor. Furthermore, a smaller energy gap between the LUMO and HOMO energies has a considerable influence on the intermolecular charge transfer and bioactivity of molecules. Thus, a wide energy gap observed in the hit molecules negatively affect the electron to move from the HOMO to the LUMO, which subsequently led to a weak affinity of the inhibitor for COVID-19 main protease. The E_gap_ value decreases according to the following**:** oxacillin (0.1021eV) **>** podophyllotoxin (0.0145eV) > gefitinib (0.0351eV) > simvastatin (0.0230eV) > lovastatin (0.0145eV). Hence, the reactivity order increases according to: oxacillin (0.1021eV) **>** podophyllotoxin (0.0410eV) > gefitinib (0.0351eV) > simvastatin (0.0230eV) > lovastatin (0.0145eV) where the most reactive is clearly lovastatin (0.0145eV). The order of reactivity increases conforms with the decreases in energy gap values. The chemical potential (μ) indicate negative values for all the compounds, which implies good stability, and the formation of a stable complex with the receptor. Also, lovastatin and simvastatin have the least hardness values (**η)**, among the hit molecules and which correlated with the trend of molecular docking (Table 3).

**Table 3:** Frontier molecular orbital energies (eV) and global reactivity descriptors.

Entry	Zinc ID	E (I)	E (A)	E (A-I)	η	μ	Ω
1	Gefitinib	-0.1427	-0.1076	0.0351	0.0176	-0.1252	0.4453
2	Oxacillin	-0.1503	-0.0482	0.1021	0.0601	-0.0993	0.0820
3	Lovastatin	-0.1930	-0.1785	0.0145	0.0072	-0.1858	2.3973
4	Podophyllotoxin	-0.1213	-0.0803	0.0410	0.0070	-0.1008	0.7258
5	Simvastatin	-0.2142	-0.1912	0.0230	0.0115	-0.2027	1.7864

**Figure 11:**
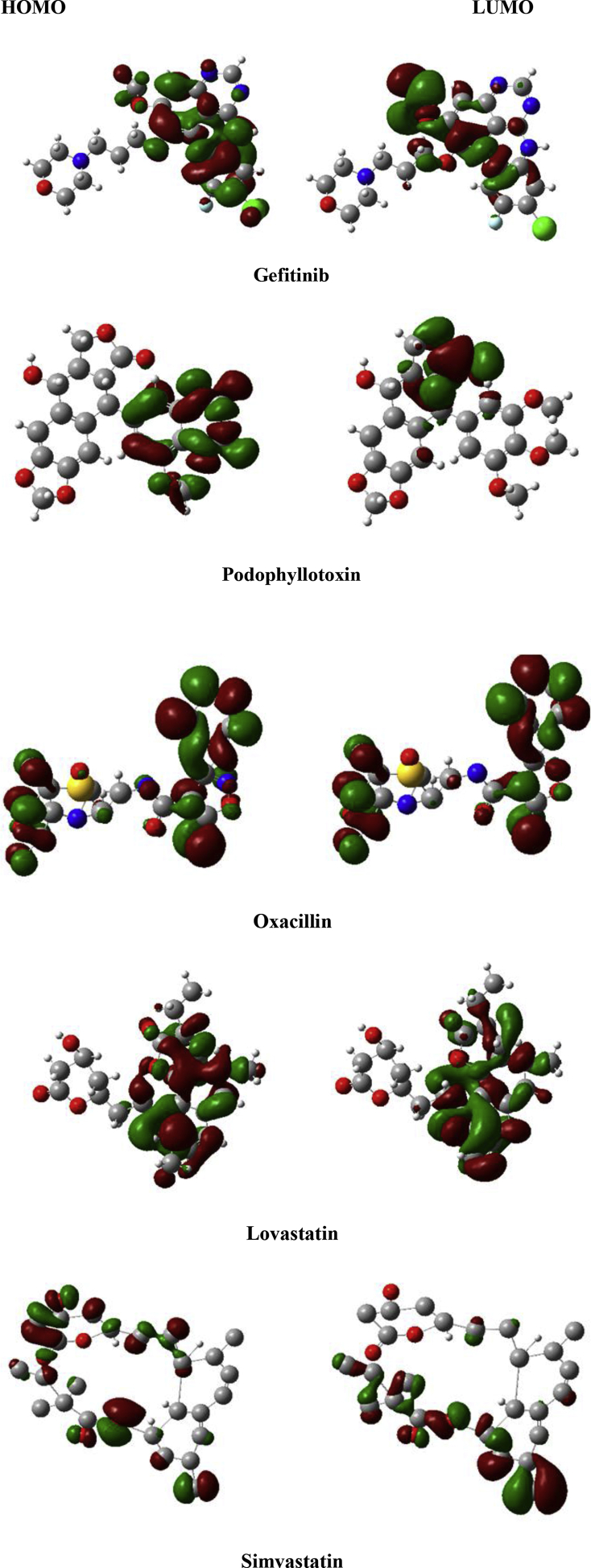
HOMO and LUMO plots of the hit compounds.

The graphic results from DFT calculations are presented in Figure 11. The red and green parts represent the cloud density of frontier orbital at HOMO or LUMO states. Finally, by comparing the values of molecular orbital energies (eV), global reactivity descriptors, and binding affinities value of five-hit compounds, simvastatin, and lovastatin may be considered as potential COVID-19 main protease inhibitors.

**Figure 12:**
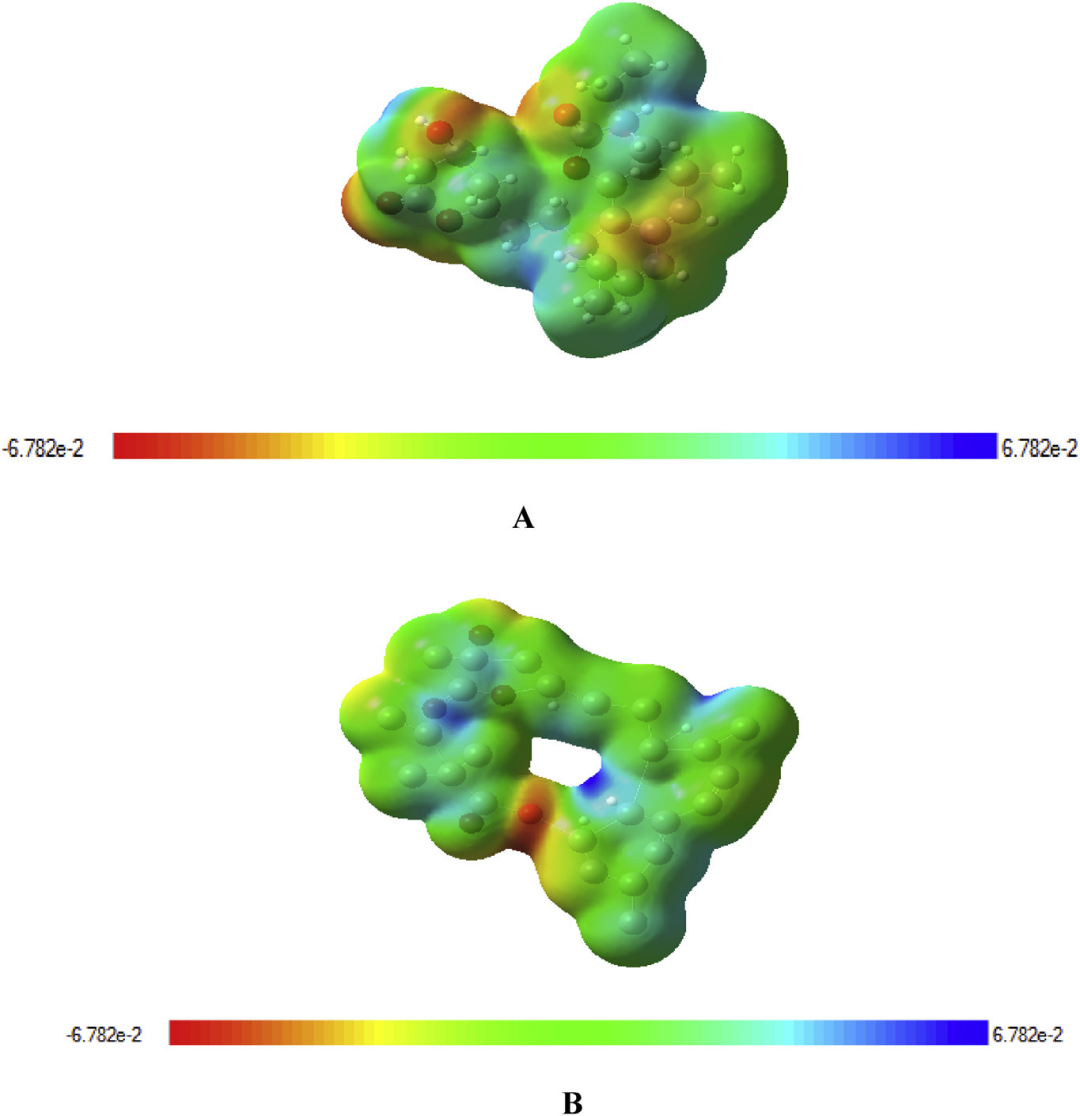
Molecular electrostatic surfaces (A) simvastatin and (B) lovastatin.

The molecular electrostatic potential (MEP) surface provides details about charge distribution and also predicts reactive sites for electrophilic and nucleophilic attack in a compound. Meanwhile, the MEP surface of simvastatin and lovastatin were evaluated using the DFT/b3lyp method as shown in Figure 11 most negative regions are shown by red, most positive regions in blue, and zero potential regions are visualized in green. MEP mapped surface of simvastatin range from −0.0678 a.u (deepest red) to 0.0678 a.u (deepest blue) and lovastatin range from −0.0672 a.u (deepest red) to 0.0672 a.u (deepest blue) Figure 12.

## 4 Conclusion

In this research paper, virtual screening was successfully used to identify five new FDA approved candidate molecules similar to the efavirenz scaffold, and their binding affinity was superior to the native ligand in the active pocket of the COVID-19 main protease, i.e.; podophyllotoxin, oxacillin, lovastatin, simvastatin, and gefitinib. The re-docked results after MD simulation revealed an increase in the binding affinity of simvastatin and lovastatin (8.2 and 7.9 kcal/mol), respectively. Notably, there was a decrease in the binding affinity of podophyllotoxin (7.7 kcal/mol), and the binding affinity of oxacillin and gefitinib (7.7 kcal/mol) remained constant. The docking results showed that H-bonds and hydrophobic interactions might play important roles in contributing to the molecular interactions between the active compounds and the COVID-19 main protease. The DFT calculations and molecular docking showed that lovastatin and simvastatin may be considered as potential hits as anti-coronavirus agents and can be selected for further studies like modification of the scaffold, characterization, and *in vitro* evaluation. The predicted physiochemical and ADMET parameters were within the acceptable optimal requirements for drug development.

## Declarations

### Author contribution statement

Maryam Amra Jordaan: Conceived and designed the experiments; Analyzed and interpreted the data; Wrote the paper.

Oluwakemi Ebenezer: Performed the experiments; Analyzed and interpreted the data; Wrote the paper.

Nkululeko Damoyi, Michael Shapi: Analyzed and interpreted the data; Contributed reagents, materials, analysis tools or data.

### Funding statement

This research did not receive any specific grant from funding agencies in the public, commercial, or not-for-profit sectors.

### Competing interest statement

The authors declare no conflict of interest.

### Additional information

No additional information is available for this paper.
